# Exploring the Information Needs of People With Elbow Osteoarthritis Seeking Healthcare: A Qualitative Interview Study

**DOI:** 10.1002/msc.70135

**Published:** 2025-06-04

**Authors:** Katy Boland, Maria Moffatt, Chris Littlewood

**Affiliations:** ^1^ Wrightington, Wigan and Leigh Teaching Hospital NHS Foundation Trust Wigan UK; ^2^ School of Health & Society University of Salford Salford UK; ^3^ School of AHPs and Nursing University of Liverpool Liverpool UK

**Keywords:** elbow osteoarthritis, patient information, qualitative study, self‐management, shared decision making

## Abstract

**Objective:**

To explore the information needs of people with elbow osteoarthritis.

**Design:**

Qualitative interview study using reflexive thematic analysis.

**Setting:**

A single National Health Service Teaching Hospital Trust and associated primary care services, providing musculoskeletal care across the clinical pathway. Interviews were conducted in person, by phone or video call according to participant preference.

**Participants:**

Twelve adults with clinically diagnosed elbow osteoarthritis, under the care of a general practitioner or consultant elbow surgeon, were included.

**Results:**

Four themes were developed: (1) self‐management in action, (2) experience of treatment options and navigating surgical decision making, (3) negotiating uncertainty and (4) active information seeking. Participants experiences were wide ranging and their varied information needs were at times unmet, particularly when related to treatment options, prognosis and surgical decision making. Across the clinical pathway, information was reported by some to be unclear or contradictory. Participants discussed a range of preferences for information sources. Accessing information was challenging for some participants and various barriers were discussed.

**Conclusions:**

This is the first study to report the lived experience of people with elbow osteoarthritis and their information needs. For some, accessing information can be challenging, and the unmet information needs can affect the ability to self‐manage ongoing symptoms and participate in treatment decisions. These findings provide a platform for the development of accessible, meaningful and culturally sensitive information sources capable of contributing to optimal treatment pathways.

## 1 Introduction

Osteoarthritis is a common, long‐term condition that causes joint pain and stiffness. Elbow osteoarthritis is less common than shoulder, hip and knee osteoarthritis and as a result, little is known about the experiences of people with this condition. However, elbow osteoarthritis can have a significant effect on a person's quality of life, including their ability to complete essential day‐to‐day tasks such as eating, washing, dressing, sleeping, working and undertaking leisure activities (Adla and Stanley 2011). Optimal treatment pathways have not been established for people with osteoarthritis of the elbow (Singh et al. 2022).

High quality, reliable and accessible information supports people to be actively involved in decisions about their health care and to self‐manage their symptoms. Clinical guidelines reflect the importance of information provision (National Institute for Health and Care Excellence (NICE) 2022), yet people with osteoarthritis consistently report a need for more information, communicated more clearly and from a wider range of sources (Chou et al. 2018; Sotthivej et al. 2020).

Information that can be individualised, is more accessible and addresses common misconceptions has the potential to improve clinical outcomes for people with elbow osteoarthritis and contribute to the development of optimal treatment pathways. Such information provision could support patients to actively participate in decision making, treatment choices, and self‐management (National Institute for Health and Care Excellence (NICE) 2021). Therefore, this study aims to explore the experience and information needs of people with elbow osteoarthritis to identify what information is needed and when.

The study objectives are as follows:

1. To understand the experience of people with elbow osteoarthritis and their information needs.
2. To understand how people with elbow osteoarthritis use existing information to make decisions about managing their condition.
3. To explore the extent to which the information needs of people with elbow osteoarthritis differ across the treatment pathway.

## 2 Methods

This qualitative study was undertaken in the context of a pragmatist theoretical framework and is reported in accordance with the consolidated criteria for reporting qualitative research (Tong et al. 2007) (Supporting Information S1).

The sponsor NHS Trust's Patient and Public Involvement group provided valuable contributions to the study prior to approvals, specifically reviewing patient facing study documents, the proposed recruitment strategy and lay summary. The group agreed that ‘information’ was an acceptable term to use and meant the same thing to most people. Changes made following this meeting included the use of ‘informal conversation’ rather than interviews, clarification of where participants would be recruited from and alternative options for participants to ‘opt in’ to the study, with less reliance on potential participants being able and willing to utilise QR codes.

Recruitment took place between May and November 2024 across a single National Health Service Teaching Hospital Trust and associated primary care services in the Northwest of England. This organisation provides musculoskeletal services across primary, secondary, and tertiary care settings, enabling recruitment of participants with a broad range of experiences across the care pathway.

Adults, with clinically diagnosed elbow osteoarthritis, registered with a general practitioner or under the care of a consult orthopaedic elbow surgeon at the Trust were eligible to take part. Individuals with a level of written and spoken English that would prevent their participation in interviews were excluded. Purposive sampling was used with reference to gender and employment status. These characteristics were selected because of known differences: gender in terms of prevalence (Nakayama et al. 2022; Stanley 1994) and information seeking behaviours (Manierre 2015) and employment status in terms of the functional demand placed on the elbow (Burden et al. 2024; Cheung et al. 2008; Samdanis et al. 2020).

Potential participants under the care of consultant elbow surgeons were screened and identified from both orthopaedic outpatient clinic appointments and lists of existing patients. In addition, first contact physiotherapy clinics, acting as participant identification centres, screened lists of existing patients. Potential participants were either introduced to the study by their clinician or received a postal study invitation letter and participant information leaflet. For potential participants referred to the lead author (KB) by the clinical team or returning a competed consent to contact form, a follow‐up phone call from KB provided opportunity to provide more information and to have questions about the study answered. Potential participants agreeing to proceed were invited to return a signed consent form prior to a mutually convenient interview time and date being agreed. Ongoing consent for participation was confirmed verbally and audio recorded at the start of each interview.

KB is a white, female physiotherapist and clinical academic, with a clinical and research interest in people with elbow osteoarthritis and formal training in qualitative interview techniques. KB undertook all interviews and was unknown to all participants. It was made clear during the interviews that whilst KB is a member of the clinical team at the Trust, she was not working in this capacity during study activities. The confidential nature of the interview was highlighted in patient facing study material and during the follow‐up phone call and consent conversation. Whilst KB is an established clinician and familiar with the subject area, she is a less experienced interviewer and is supported by established academics with qualitative experience.

Sample size was determined using the principle of information power (Malterud et al. 2016) rather than data saturation. Considering the narrow study aim, use of purposive sampling to increase sample specificity and availability of existing literature regarding information needs in people with osteoarthritis in other joints, a sample size of up to 15 participants was proposed. To determine the quality of the data, the study team reviewed data from the first 10 interviews and early code generation, with a further two interviews being carried out to ensure that the codes appropriately captured important meaning across the dataset. At this stage, it was determined through critical discussion within the research team that sufficient data had been collected to enable the study objectives to be met.

Data were collected using individual semi‐structured interviews offered in the patient preferred format: virtually using Microsoft Teams, by telephone or in person at the NHS Trust's clinical research hub which is situated in the local community. A predetermined, but flexible topic guide (Supporting Information S1) was used to steer the conversations, developed with input from the study team and informed by a review of current literature. Changes made during piloting of the topic guide included additional prompts and rewording questions to make the language simpler. Interviews were scheduled to last up to 60 min. Telephone and in person interviews were audio recorded, and Microsoft Teams interviews audio and video recorded.

Audio data were transcribed in an intelligent verbatim format by KB with filler words and repetition omitted as they were not seen to add to or change meaning (Cohen et al. 2002). Additional data from handwritten field notes made during the interviews were included in these transcripts. Data were initially analysed by KB, with Braun and Clarke’s (2022) 6‐stage model of thematic analysis, used to explore patterns of meaning across the dataset. Regular discussion across the team and use of a reflexive journal supported the recursive process of analysis which occurred alongside ongoing data collection. To meet the study objectives, analysis focused on participants perspectives and lived experience, approaching the data with an inductive orientation and semantic focus. Microsoft Word & Excel (Microsoft 365) were used to manage the data. Participant checking of transcripts was not undertaken. Data familiarisation involved the lead researcher reading and re‐reading all transcripts in their entirety before proceeding to initial code generation. Three rounds of coding by the lead researcher and iterative reflexive discussion across the team generated a list of 28 initial codes which were further refined to 21. These codes were then combined into candidate themes which, after further revision against both coded extracts and the full dataset by the study team, were mapped and developed into the themes presented here.

## 3 Results

Sixty‐six patients were screened as potentially eligible and invited to participate in the study. Of these, 28 provided consent to contact and were approached. Of the 10 individuals who then declined participation, one stated a lack of time, four did not provide a reason and five were unable to be contacted. An additional six potential participants who initially provided consent for contact were not contacted in line with the purposive nature of the sampling.

Twelve participants consented to take part in interviews. Participant characteristics are shown in Table 1. A single interview took place in person, one via video call and the remainder by telephone. Interviews ranged in duration from 19 to 42 min, with the average being 28 min.

**TABLE 1:** : Participant characteristics.

Participant	Age (years)	Gender	Employment status
P01	> 65	Male	Not working
P02	> 65	Female	Not working
P03	45–65	Female	Not working
P04	> 65	Female	Not working
P05	45–65	Female	Not working
P06	< 45	Male	Not working
P07	> 65	Male	Not working
P09	45–65	Female	Working
P10	45–65	Male	Working
P12	> 65	Female	Not working
P13	45–65	Female	Not working
P14	45–65	Male	Not working

Four themes were developed and are reported below with pseudonymised data extracts used to illustrate them.

### 3.1 Theme One: Self‐Management in Action

Participants described the changes they made which enabled them to continue doing the activities that mattered to them, despite their symptoms.

I think you’ve got to use your common sense. And just do your best… Gradually you've got to change all these things to make life easier for yourself. But not to just opt out of doing them, you know.

(P04)

Changes included learning to use their non‐dominant hand, getting help from family and friends, spreading tasks throughout the day or taking regular breaks, choosing lighter or adapted domestic appliances and use of assistive equipment or aids.

Taught myself to write with my left hand while I was holed up at first… I feel I use my left hand more than my right these days.

(P06)

Four participants also described experiencing a variety of changes to their employment, including ill health retirement, making alternative career plans and changes to their usual duties. Manual work was described as particularly challenging.

My job at the time, you’re heavy lifting and I couldn’t lift. I was having to use my knees, you know your dropping everything, couldn’t lift anything heavy.

(P10)

One participant spoke openly about the trauma of the workplace accident preceding the onset of their symptoms.

I couldn't do that job what I was doing. I mean, I didn’t really want to finish, I’d done 16 years all together but it wasn’t just that, it was the trauma of it as well, I couldn’t go back in there again. It was all that… I mean, I’m < 60 s> now, I wasn’t hoping to finish work so soon.

(P13)

Participants appeared to make these changes without instruction, talking explicitly about having to teach themselves, figure things out and adapt to keep going.

But I think it comes to its just common sense, isn't it. That's it, it’s just common sense.

(P10)

Despite these adaptations, a wide range of symptoms were still described by participants including pain, the elbow joint locking, being unable to use the arm to weight bear, joint stiffness, weaker grip and pins and needles in the hand. Participants discussed the negative effect these symptoms had on their sleep, mobility and confidence, general activity levels, socialising and various aspects of day‐to‐day function.

Yeah, its traumatising really, it's like just having that confidence. I use a stick as well now, just because if I fall, I can't even stop myself from my arm. I couldn't even get up off the floor if I fall down.

(P13)

You're constantly using your arm, it’s my right hand, my right elbow. So it just affects everyday things.

(P09)

I’ve given up driving, to be honest. You have to give up all sorts…

(P04)

### 3.2 Theme Two: Experience of Treatment Options and Navigating Surgical Decision Making

Participants discussed experiencing a range of treatments across various health care settings. Pain medication, injections and physiotherapy were described as providing temporary relief from symptoms and having limited effectiveness.

I take plenty of painkillers. But like, you can't live off them permanently. Cos your body gets used to them and then it doesn't work then after a while.

(P03)

I've had injections, don’t really seem to do much really. That was it. I think I’ve had two or three across both elbows. They don’t really seem to do much.

(P10)

Cause the physio’s keeping me going at the moment, I don’t know how long it will go on for. If they stop that at the same time as < clinical follow up > stops, I'm back to square one. If there’s no change then, you know what I mean?

(P13)

One participant was explicit in their description of the long‐term nature of osteoarthritis and the lack of a ‘cure’.

Everything I found is once you've got it, you've got it. There's no going back.

(P14)

The uncertainty around predicting outcomes after surgery was described by two participants with a sense of surgery being a last resort. Participants' descriptions of surgical decision‐making included explicit requests for more information and support particularly relating to risks and benefits and prognosis.

So, at this point there's not enough advantages to take the risks for me… but it’s a fine balance, it always is isn't it. So I’d want somebody again, to help me to weigh up that balance.

(P05)

I know it's elective surgery and I know I have to make the decision, but I suppose I feel like I need a stronger message about we can do this, but actually you might be better just putting up with it or we can do this and actually you'll be in a better place at the end of it. And I don't know if that's an ethical thing for a doctor to opt to say [laughing]…

(P02)

### 3.3 Theme Three: Negotiating Uncertainty

When participants described their experiences of receiving a diagnosis, at times the information was subject to change or at odds with how participants understood and experienced their symptoms.

He's very clear, you know, it is arthritis that is your problem. It's not tennis elbow, but there's no doubt that the tendons in my arm have been damaged.

(P02)

It was just a bit like, it's just the norm for it to be a tennis elbow and I did say to her [GP] at the time it doesn't feel like anything like that, it feels different. I’m getting this sharp pain and I want to know what’s causing it. That’s when she sent me for an Xray…

(P09)

Other participants discussed how their osteoarthritis diagnosis was implied rather than explicitly communicated.

The consultant said that I had a bit of wear in the elbow already and that when I had the operation, in the future I would get arthritis in it. But I wasn't aware that it was starting to set in already. I do get like, pain at it. Pain from the elbow as it is, that probably explains that! [laughing]. (P06)

Where participants had met multiple clinicians across the pathway, earlier information was on occasion explicitly labelled as incorrect or was contradicted.

<Surgeon> said you shouldn't. The doctor shouldn't have drained it.

(P01)

So I got referred to see <surgeon> and he said, “you’ve got arthritis, I know that without even looking at your X‐ray”. And I said well how did he [physiotherapist] not know that

(P10)

He's [surgeon] very opposed to more steroid injections because they don't last very long. Although the one that I had, I think it helped me for about 2 years before it got worse again.

(P02)

Some participants disclosed clear, ongoing information needs about treatment options and prognosis.

I just need to know what I can do to make it better.

(P09)

I don't know whether ultimately, you know this, my elbow is gonna get to a point where it's so bad that we have to look at elbow replacement, I have no idea.

(P05)

### 3.4 Theme Four: Active Information Seeking

Participants described the barriers they experienced when trying to meet their information needs across a range of settings. These included perceived delays to their care pathway whilst waiting for appointments or test results, limited time during interactions with clinicians, a lack of visible information leaflets and information not being relevant or specific to elbow problems.

And when you're in a consultation with someone you know is very busy, it's difficult to take time up asking questions about things.

(P02)

I just think it's a bit overwhelming and there's a lot of information out there. And choosing the right information from a patient perspective is difficult…

(P05)

When discussing online information, four participants disclosed that they do not access online information either by preference or lack of access. Relatives might step in to support in this case.

It might be on the Internet but a lot of elderly people don’t have computers… But if I mention to him [son] there’s a site that you can look out for he’ll definitely go on and try to find out more about it.

(P12)

Other participants used online information but were aware of the challenges including the source, reliability and relevance of the information.

A little bit of knowledge is a bad thing. And on the Internet there's a lot of rubbish, a lot misinformation. And different countries have different ways of looking at things. And different rules regarding medicine and healthcare.

(P07)

I would only go to a trusted site. If it wasn’t an authorised charity or something I wouldn’t look at it.

(P04)

One participant described their preference for supplementing the information received in person during appointments with images and videos of surgery online.

I mean, if they offer information I'll take it on board and then probably forget about it as I go through the door. [both laughing]… So going home and then hitting the internet… and if you want visuals, then YouTube is the place to go.

(P14)

Others discussed accessing online peer support, and one participant highlighted the potential for information in this form to be quite negative. Others found the groups helpful.

Some of these groups, if you go on to any of these and I haven't done this for elbows, but you can scare yourself witless sometimes.

(P05)

The Facebook groups pretty good, because people are asking different things, what they thought… you can read what other people are going through.

(P09)

## 4 Discussion

This study aimed to explore the lived experience and information needs of people with elbow osteoarthritis. Participants reported wide ranging experiences and a subsequent spectrum of information needs. Self‐management strategies were described which allowed continued participation in meaningful activities. Pain medication, injections and physiotherapy often provide only temporary relief from symptoms. When making decisions about surgical treatments, participants described explicit information needs related to risks and expected outcomes. Across the clinical pathway, information about diagnosis and management options was unclear or contradictory for some participants, leading to additional unmet information needs. Accessing information could be challenging, with some participants describing barriers including time and a lack of relevant and trustworthy information, particularly online. Others expressed a preference for peer support groups or used online videos to meet their information needs.

The symptoms experienced by participants, including elbow pain and joint stiffness, are widely recognised (Cheung et al. 2008); however, this study calls attention to the far‐reaching and substantial impact that living with elbow osteoarthritis has on participants function, recreation and work. These findings reflect the experiences of people with osteoarthritis in other joints (Smith et al. 2014; Toye et al. 2022). The model of supported self‐management proposed by the National Health Service (National Health Service, n.d.), where people work with clinicians to develop the knowledge, skills and confidence to manage their long‐term condition optimally, did not appear to feature strongly in the experiences of these participants during health care visits. There also appears to be little consideration by participants of the broader lifestyle or behavioural changes recommended by clinical guidelines in the management of osteoarthritis (National Institute for Health and Care Excellence (NICE) 2022).

The optimal management of elbow osteoarthritis remains unclear. Research priorities include understanding which non‐operative treatments are most effective, expected surgical outcomes and what works best for younger people (Singh et al. 2022). The findings of this study reflect this uncertainty, with non‐surgical treatments perceived as unacceptable long‐term management options for some participants. A previous scoping review of patients perceived needs of osteoarthritis health information (Chou et al. 2018) highlights patients' dissatisfaction with both the delivery and content of health information in a population predominantly diagnosed with hip or knee osteoarthritis. In line with previous research, participants in this study discussed an explicit need for more information about treatment options, particularly the risks, benefits and expected outcomes of surgical procedures, to support the decision‐making process. Whilst some participants had chosen not to proceed with surgery, others were undecided. The model of shared decision‐making proposed by the (National Institute for Health and Care Excellence (NICE) 2021) was not always fully evident in participants' descriptions of their experiences. Whilst interventions such as decision support tools can aid decision making for people with osteoarthritis (Bozic et al. 2013), no such tool exists currently for people with elbow osteoarthritis.

This study highlights the experiences of some participants who received contradictory information across the clinical pathway regarding their diagnosis or previous treatments. Underutilisation of core components of the clinical guidelines for the management of osteoarthritis is widely reported. Implementing models of osteoarthritis care is complex, with a range of barriers (Swaithes et al. 2020). The complexity of treatment decision making for osteoarthritis is also well recognised (Barker et al. 2023), where conflicting opinions and inconsistent information complicate decision making for patients. Additionally, conflicting information can drive information seeking behaviours in people with osteoarthritis, alongside other decision‐making strategies (Elstad et al. 2012), potentially stimulating a cycle of increasing dependence on clinical experts as information sources. Some participants in this study also experienced the diagnosis of elbow osteoarthritis being implied rather than explicitly communicated, potentially delaying participant information seeking behaviours. Whilst avoidance of medical jargon and language with negative emotional associations such as ‘degenerative changes’ are promoted when working with people with osteoarthritis (Barker et al. 2014), supporting people to understand their diagnosis is an essential first step in managing symptoms.

Participants in this study experienced barriers to their information needs associated with navigating the health care system including time during and between appointments and whilst waiting for test results. Online information seeking was prevalent among these participants, in line with previous research findings (Sotthivej et al. 2020). However, some participants chose not to or were unable to access online information which is an important consideration when developing information resources. People with osteoarthritis access information from a range of sources including the internet, support groups, family and friends and clinicians (Chou et al. 2018). Health literacy levels may influence the amount and source of information that people with osteoarthritis access, with those with higher health literacy seeking more information online (Ellis et al. 2012). However, publicly available information for people with osteoarthritis may not reflect clinical guidelines (Goff et al. 2023; Lalande et al. 2024) leading to potential misinformation and misconceptions.

Whilst this study aimed to explore the experiences of people with elbow osteoarthritis across the clinical pathway, a limitation involved the recruitment of only a single participant from the primary care setting. The majority of participants were further along the treatment pathway and therefore more reliant on recall of their experiences in the earlier stages of their clinical journey. The majority of participants were therefore recruited from the hospital where KB works clinically. The choice of reflexive thematic analysis, involving regular use of a reflexive journal and meetings with the research team, who are independent of this setting, allowed KB to use her identity as being part of the team to enrich the analysis. An additional limitation relates to the interview topic guide, which was based on a review of existing literature and piloted among clinicians. The semi‐structured nature of the interviews allowed some flexibility for deeper exploration of topics; however, the lack of patient and public involvement in developing this guide may have resulted in meaningful aspects of the lived experience of participants not being explored. Future work could also consider the experiences of other stakeholders such as clinicians, managers, funders and academics relating to identifying and meeting the information needs of people with elbow osteoarthritis.

## 5 Conclusion

This is the first study to report on the lived experience of people with elbow osteoarthritis and their information needs, which at times are unmet. For some, accessing information can be challenging, and unmet information needs can affect the ability to self‐manage ongoing symptoms and participate in decisions about healthcare. In particular, this study highlights limitations to accessing online information for some people with elbow osteoarthritis, relating both to preference and digital exclusion. Overall, this study strengthens the idea that people with osteoarthritis need more information. These findings provide a platform for the development of accessible, meaningful and culturally sensitive information sources capable of contributing to optimal treatment pathways.

## Author Contributions

**Katy Boland:** conceptualization, formal analysis, investigation, methodology, project administration, writing – original draft preparation, writing – review and editing. **Maria Moffatt:** conceptualization, methodology, supervision, validation, writing – review and editing. **Chris Littlewood:** conceptualization, methodology, supervision, validation, writing – review and editing.

## Ethics Statement

Ethical approval was obtained from the South Central–Berkshire Research Ethics Committee (Ref: 24/SC/0088).

## Conflicts of Interest

The authors declare no conflicts of interest.

## Supporting information

Supporting Information S1

## Data Availability

Due to the nature of the ethical approval, supporting data are not available for sharing beyond the research team.
