# Supplementary material for: Exploring the Information Needs of People With Elbow Osteoarthritis Seeking Healthcare: A Qualitative Interview Study
Source: Musculoskeletal Care. 2025 Jun 4;23(2):e70135. doi: 10.1002/msc.70135 (PMC12138164; doi:10.1002/msc.70135)
Supplement: Supplementary file 1 — Supporting Information S1 [file MSC-23-e70135-s001.docx]

**Supplemental File 1-COREQ Checklist**

| **Topic** | **Item No.** | **Guide Questions/Description** | | | **Reported on Page No.** |
| --- | --- | --- | --- | --- | --- |
| **Domain 1: Research team and reflexivity** |  |  | | |  |
| *Personal characteristics* |  |  | | |  |
| Interviewer/facilitator | 1 | Which author/s conducted the interview or focus group? | | | 5 |
| Credentials | 2 | What were the researcher’s credentials? E.g. PhD, MD | | | 5 |
| Occupation | 3 | What was their occupation at the time of the study? | | | 5 |
| Gender | 4 | Was the researcher male or female? | | | 5 |
| Experience and training | 5 | What experience or training did the researcher have? | | | 5 |
| *Relationship with participants* |  |  | | |  |
| Relationship established | 6 | Was a relationship established prior to study commencement? | | | 5 |
| Participant knowledge of the interviewer | 7 | What did the participants know about the researcher? e.g. personal goals, reasons for doing the research | | | 5 |
| Interviewer characteristics | 8 | What characteristics were reported about the inter viewer/facilitator? e.g. Bias, assumptions, reasons and interests in the research topic | | | 5 |
| **Domain 2: Study design** |  |  | | |  |
| *Theoretical framework* |  |  | | |  |
| Methodological orientation and Theory | 9 | What methodological orientation was stated to underpin the study? e.g.  grounded theory, discourse analysis, ethnography, phenomenology, content analysis | | | 4 |
| *Participant selection* |  |  | | |  |
| Sampling | 10 | How were participants selected? e.g. purposive, convenience, consecutive, snowball | | | 4-5 |
| Method of approach | 11 | How were participants approached? e.g. face-to-face, telephone, mail, email | | | 5 |
| Sample size | 12 | How many participants were in the study? | | | 7 |
| Non-participation | 13 | How many people refused to participate or dropped out? Reasons? | | | 7 |
| *Setting* |  |  | | |  |
| Setting of data collection | 14 | Where was the data collected? e.g. home, clinic, workplace | | | 6 |
| Presence of nonparticipants | 15 | Was anyone else present besides the participants and researchers? | | | 6 |
| Description of sample | 16 | What are the important characteristics of the sample? e.g. demographic data, date | | | 8 |
| *Data collection* |  |  | | |  |
| Interview guide | 17 | Were questions, prompts, guides provided by the authors? Was it pilot tested? | | | 6 |
| Repeat interviews | 18 | Were repeat inter views carried out? If yes, how many? | | | 6 |
| Audio/visual recording | 19 | Did the research use audio or visual recording to collect the data? | | | 6 |
| Field notes | 20 | Were field notes made during and/or after the interview or focus group? | | | 6 |
| Duration | 21 | What was the duration of the inter views or focus group? | | | 8 |
| Data saturation | 22 | Was data saturation discussed? | | | 6 |
| Transcripts returned | 23 | Were transcripts returned to participants for comment and/or  correction? | | | 6-7 |
|  | | |  |  |  |
| **Domain 3: analysis and findings** |  |  | | |  |
| *Data analysis* |  |  | | |  |
| Number of data coders | 24 | How many data coders coded the data? | | | 6-7 |
| Description of the coding tree | 25 | Did authors provide a description of the coding tree? | | | 6-7 |
| Derivation of themes | 26 | Were themes identified in advance or derived from the data? | | | 6-7 |
| Software | 27 | What software, if applicable, was used to manage the data? | | | 7 |
| Participant checking | 28 | Did participants provide feedback on the findings? | | | 7 |
| *Reporting* |  |  | | |  |
| Quotations presented | 29 | Were participant quotations presented to illustrate the themes/findings?  Was each quotation identified? e.g. participant number | | | 8-14 |
| Data and findings consistent | 30 | Was there consistency between the data presented and the findings? | | | 8-14 |
| Clarity of major themes | 31 | Were major themes clearly presented in the findings? | | | 8-14 |
| Clarity of minor themes | 32 | Is there a description of diverse cases or discussion of minor themes? | | | 8-17 |

Developed from: Tong A, Sainsbury P, Craig J. Consolidated criteria for reporting qualitative research (COREQ): a 32-item checklist for interviews and focus groups. *International Journal for Quality in Health Care*. 2007. Volume 19, Number 6: pp. 349 – 357

**Supplemental File 2-TOPIC GUIDE**

[before recording]

**Intro:**

- Introduce myself (clinical physio working with people with shoulder and elbow problems, researcher).
- Thank participant for their time.
- Review research question (clarify ‘information’) and PIS (invited as have elbow OA), outline length of interview (60 minutes). Anonymity.
- Explain process: As we are talking I will ask questions to guide the conversation, I’ll make notes, may ask for more detail. No right or wrong answer and both positive and negative views are important.
- Confirm consent including to record.

[start recorder]

Verbally confirm ongoing consent to participation

1. Warm up:

To start off with, tell me about how you found out that you have elbow osteoarthritis?

- How did your symptoms start?
- And how long ago?
- How were you diagnosed and what investigations did you have?
- Which health care professionals were involved?

1. When you found out you had elbow osteoarthritis, what did you first want to know?

- Did you have any previous experience of osteoarthritis?
- Sometimes people find there isn’t enough information about elbow arthritis, what has your experience been?

1. So you have said that you knew enough / wanted to know more. How did that make you feel?

- Did you feel in control of your symptoms?
- And how would you describe the relationship with the health care professionals involved in your care?

1. And since your diagnosis, what have you done to find out more?

- Can you give an example?
- Have you spoken to friends or family?
- Or looked for your own information?
- Have you had to look online?

1. What has it been like for you, finding the information you need?

- How much time and effort have you used?
- Was the information you found worth it?
- How reliable have you found online information?

1. You said earlier you have had elbow arthritis for XX months / years. How has what you have wanted to know changed over this time?

- Did you want to know everything about elbow arthritis at once?
- Has where you get information from changed?
- Have you needed to find out more as time has gone on or your symptoms have changed?
- So what you have needed to know has changed. Could you give me an example of when this happened?

1. Was there a particular time since you started with symptoms that it would have helped to have more information, could you tell me why this was?

- What do you do with this information?
- And how were you feeling at the time?

1. People with arthritis sometimes tell us they prefer information that meets their individual needs. What are your thoughts about this?

- Was it individualised?
- When you spoke to XXHCPXX could they answer your questions or provide more information if needed? (expertise)

1. Closing question:

- And finally, what advice would you give someone who wants to find out more about elbow arthritis?

Wrap up:

- That’s all the questions I have for you.
- Thanks again.
- Is there anything else you would like to add that we haven’t discussed?
- Any questions for me?

[turn off recorder]

Closing:

- Thanks to the participants
- Discuss travel or parking costs / compensation as necessary
- What happens next (single interview, review transcripts, sent final report if opted in.
